# Gender and social mobility modify the effect of birth weight on total and central obesity

**DOI:** 10.1186/s12937-017-0260-7

**Published:** 2017-06-26

**Authors:** Juliana Rombaldi Bernardi, Marcelo Zubaran Goldani, Tanara Vogel Pinheiro, Luciano Santos Pinto Guimarães, Heloisa Bettiol, Antônio Augusto Moura da Silva, Marco Antônio Barbieri

**Affiliations:** 1 Departamento de Nutrição, Universidade Federal do Rio Grande do Sul, Av. Jerônimo de Ornelas, 721 - Santana, 90040-341 Porto Alegre, Rio Grande do Sul Brazil; 2 Departamento de Medicina, Programa de Pós-Graduação em Saúde da Criança e do Adolescente, Universidade Federal do Rio Grande do Sul, Rua Ramiro Barcelos, 2400, Santana, 90035-003 Porto Alegre, Rio Grande do Sul Brazil; 3 Unidade de Bioestatística, Grupo de Pesquisa e Pós-graduação, Hospital de Clínicas de Porto Alegre, Rua Ramiro Barcelos, 2350, Santa Cecilia, 90035-903 Porto Alegre, Rio Grande do Sul Brazil; 4 Departamento de Puericultura e Pediatria, Faculdade de Medicina de Ribeirão Preto, Universidade de São Paulo, Av. Bandeirantes, 3900 - Monte Alegre, Ribeirão Preto, São Paulo 14049-900 Brazil; 5 Departamento de Saúde Pública, Universidade Federal do Maranhão, Av. dos Portugueses, 1966 - Vila Bacanga, MA, 65085-580 São Luís, Maranhão Brazil

**Keywords:** Early life programming, Birth weight, Social transition, Body mass index, Waist circumference

## Abstract

**Background:**

Little is known about the interaction between gender and low birth weight (LBW) and lifelong social mobility as an explanation of the etiology of obesity. The aim of the present study was to evaluate total and central obesity according to gender, LBW and social mobility, within the context of the epidemiological transition in middle-income countries. We hypothesize that there are more pronounced metabolic consequences of social mobility for women born with LBW.

**Methods:**

We used data from a birth cohort study conducted in Ribeirão Preto, São Paulo, Brazil. Data regarding anthropometric measurements, schooling and smoking status were collected at 23-25 years of age. Social mobility was determined based on maternal and adult offspring schooling and categorized as Low-Low, Low-High and High-High. Analysis of covariance was performed to assess the association between social mobility and body mass index (BMI) or waist circumference (WC) in adulthood, stratified by LBW and gender.

**Results:**

Data on 6827 singleton pregnancies were collected at birth in 1978/79 and a sample was followed up in 2002/04. A total of 2063 subjects were included in the study. Mean age was 23.9 ± 0.7 years, 51.8% (*n* = 1068) were female and the LBW was 6.2% (*n* = 128). There was a triple interaction between social mobility, LBW and gender. Among women born without LBW, BMI and WC were higher in the Low-Low group compared to High-High schooling group. Among LBW women, BMI and WC were higher in the Low-Low group compared to the Low-High group.

**Conclusions:**

Women born with LBW belonging to the low schooling group in early adulthood had high BMI and WC, compared to the Low-High social mobility group.

## Background

A number of hypothetical models have been proposed over the last fifty years in order to explain the changes in the pattern of health and disease [1–4]. There are models proposing a perspective of epidemiological transition based on the presence of early life stress and environmental adequacy during the life course, which could influence metabolic patterns in adulthood [3, 5–8]. In addition, there are evidences that the maternal capital hypothesis, a phenotypic change dampened by maternal buffering, was mainly raised to address associations between social and health inequality [9–11]. From an evolutionary perspective, a recent study found that individuals whose mothers were young, who belonged to an ethnic minority, and who breastfed for a short period of time were more likely to be obese in adulthood, after controlling for factors present in infancy, adolescence, and adulthood [10].

Previous studies have shown that social improvement has a positive immediate influence on metabolic markers of vulnerable social groups after adjustment for potential confounders and that this influence is different for men and women [12–14]. Heraclides and Brunner, using retrospective data from a cross-sectional study of 4598 participants, observed that social mobility and social accumulation (social advantage or disadvantage) were associated with the development of overweight and obesity in adulthood, with stronger effects among women [15]. Also, a study published by our research group, in Brazil found that increase income in early adulthood was associated with a significant reduction in body mass index (BMI), waist circumference (WC) and waist-to-hip ratio only in females [16], supporting the evidence that upward social mobility may have different implications according to gender.

Brazil, like other middle-income countries, has been involved in an process of social and demographic transition with reduction of infant mortality, increase in life expectancy and improvements in social standards [17]. On the other hand, the country still has high rates of low birth weight (LBW) in infants when compared to developed countries [18, 19]. There are evidences that an individual’s birth weight, considered a strong proxy when controlling for confounders, can be a correlate of a harsh environment or vulnerable condition with respect to adult health status [20, 21]. Thus, researchers have been investigating the relationship between infant birth weight and social family determinants during childhood [22–24]. On this scenario, studies of early life stress, as indexed by LBW, followed by social mobility, could provide insights about changes in health and disease patterns, especially regarding the etiology of obesity [25, 26].

The objective of the present study was to evaluate the influence of social mobility during the life course on BMI and WC of individuals who experienced the epidemiological transition in Brazil (1978/1979 and 2002/2004) stratified by gender and birth weight.

## Methods

### Sample and participants

We analyzed prospective data from the Ribeirão Preto Cohort Study, collected in 1978/1979 and 2002/2004. Ribeirão Preto is a city of over half-a-million inhabitants in the urbanized South East of Brazil (São Paulo). The first phase of the study lasted from June, 1978 to May, 1979. A total of 9067 newborn babies were included (98% of all live births in the city over that period). Subjects who were not from Ribeirão Preto or did not reside in the city at the time of delivery (*n* = 2094) were excluded, with the final sample consisting of 6973 liveborns (6827 singletons and 146 twin pairs). The second phase of cohort follow up started in 1994 (*n* = 2846) and the third phase started in 1996/97 (*n* = 2048).

The fourth cohort follow-up was started in 2002/2004, when the individuals were 23-25 years old. From the original cohort, of 6827 singletons, 343 had died and 819 could not be located during follow-up; thus, 5665 individuals living in the same geo-economic area were contacted, corresponding to 30% of the calculated sample size of the eligible population. Updated addresses were retrieved from a number of databases including the Unified Health System (SUS) electronic database, lists of users of private health plans, school charts, and military recruitment charts. Losses to follow-up (*n* = 705) occurred because of refusal to participate, imprisonment or failure to attend an interview. These losses were replaced using the same sampling frame, resulting in 2063 young adults (*n* = 995 males and *n* = 1068 females). The protocol methodology and sample characteristics have been published elsewhere [27, 28].

### Outcomes

Anthropometric outcomes: trained staff measured weight and height, with the person barefoot and wearing light clothing. BMI was calculated by dividing weight (in kilograms, kg) by squared height (in squared meters, m^2^) [29]. Height at adult age was measured to the nearest centimeter using a wood and Formica stadiometer resistant to deformation. Weight was measured with a Filizola® scale (São Paulo, SP, Brazil) with 100 g graduations and with 140 kg capacity. WC was measured as the smallest circumference (in centimeters, cm) between the ribs and the iliac crest, while the participant was standing with the abdomen relaxed, at the end of a normal expiration.

### Covariates

Child's birth weight: was measured with appropriate devices donated by the research team to all hospitals. The babies were naked and were weighed on weekly calibrated scales with 10 g precision and classified as low birth weight (<2500 g) or not low birth weight (≥2500 g).

Social mobility: calculated by the authors using maternal schooling (in years) and adult offspring schooling (in years) at the time of the interview, was classified as low (schooling between 0-8 years) or high (schooling ≥ 9 years). Maternal schooling at birth was used as a proxy of offspring socioeconomic status (SES) during childhood. Maternal schooling was chosen due to its significance in the contemporary socioeconomic context, with current association with material and nonmaterial goods, such as access to information and behavior in the presence of health challenges, and social status [30].

The four possible classifications of social mobility were: Stable Low-Low (Low-Low group), Ascending Low-High (Low-High group), Descending High-Low (High-Low group) and Stable High-High (High-High group). The High-Low (High-Low group) category was excluded from analysis because there were only 13 individuals.

### Confounders

The model was adjusted for smoking during pregnancy (smoker and nonsmoker, determined during a postpartum interview) and gestational age (in weeks) because the variables were associated with birth weight.

### Statistical analysis

Categorical variables were expressed as absolute (*n*) and relative (%) frequencies. Continuous variables were expressed as mean, standard deviation (SD) and 95% confidence intervals (CI). The Chi-square test was performed to analyze categorical variables and analysis of variance (three way-ANOVA) to compare continuous variables, followed by the Bonferroni *post hoc* test, when indicated.

We examined the metabolic outcomes (BMI and WC) according to birth weight (LBW and no LBW), social mobility (Low-Low group, High-Low group and High-High group) and gender (female and male) calculating population averages and testing for a trend. Analysis of covariance (ANCOVA), followed by the Bonferroni *post hoc* test, when indicated, was performed by adding the factors sequentially.

Data were analyzed using the Statistical Package for the Social Sciences® (SPSS) version 18.0 software (SPSS Inc., Chicago, IL, USA). The levels of significance were set at *P* < 0.05 in all analyses.

### Ethical aspects

This cohort study was approved by the Research Ethics Committee of Hospital das Clinicas de Ribeirão Preto, Faculdade de Medicina de Ribeirão Preto, Universidade de São Paulo (protocol HCRP n. 7606/99), Brazil.

All subjects gave written informed consent to participate in the cohort study.

## Results

A total of 2063 subjects were included in the study and sample size varied for each variable. The mean (± SD) age of the young adults was 23.9 ± 0.7 years and 51.8% (*n* = 1068) were female. The LBW rate was 6.2% (*n* = 128) and 27.0% (*n* = 546) of mothers and 84.5% (*n* = 1743) of the adult offspring had high schooling (≥9 years).

The maternal and adulthood variables are shown in Table 1. LBW was significantly more common among low education mothers (*P* = 0.007; values: 83.9% *vs*. 72.3%) and among mothers who smoked any time during pregnancy (*P* < 0.001; values: 44.8% *vs*. 24.1%) when compared to non LBW. As expected, there was an association between birth weight < 2500 g and lower gestational age (*P* < 0.001; values: 34.1% *vs*. 5.7%). Individuals born with LBW had significantly less education (*P* < 0.001; values: 27.3% *vs*. 14.7%) when compared to non LBW. No other statistically significantly differences were found (*P* > 0.05).

**Table 1:** Maternal and adulthood characteristics according to birth weight, 1978/79 Ribeirão Preto Birth Cohort

Variables	Birth weight ≥2500 g (*n* = 1935)		Birth weight <2500 g (*n* = 128)		P*
	*N*	%	*n*	%	
Maternal					
Schooling (years)^a^					
High	526	27.7	20	16.1	**0.007**
Low	1373	72.3	104	83.9	
Age (years)^a^					
< 19	140	7.3	13	10.2	0.429
19-34	1622	84.3	105	82.7	
≥ 35	162	8.4	9	7.1	
Smoking during pregnancy^a^					
Nonsmoker	1441	75.9	69	55.2	**<0.001**
Smoker	457	24.1	56	44.8	
Type of delivery					
Vaginal	1314	67.9	88	68.7	0.920
Caesarean	621	32.1	40	31.3	
Gestational age (weeks)^a^					
≥ 37	1460	94.3	58	65.9	
< 37	88	5.7	30	34.1	**<0.001**
Adulthood					
Schooling (years)					
High	1650	85.3	93	72.7	**<0.001**
Low	285	14.7	35	27.3	
Gender					
Male	942	48.7	53	41.4	0.133
Female	993	51.3	75	58.6	
Smoking status					
Nonsmoker	1607	83.0	102	79.7	0.392
Smoker	328	17.0	26	20.3	

^a^ Totals may not add up to *n* = 2063 because of missing values

*P refers to the chi-squared test

Bold text: Significant differences between groups (*P* < 0.05)

**Fig. 1:**
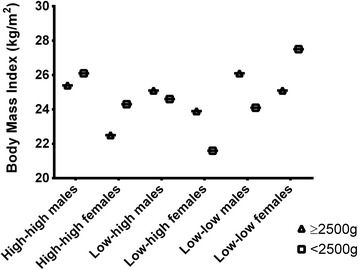
Distribution of mean BMI values (females *vs.* males) according to social mobility and birth weight

The distribution of BMI and WC according to birth weight, social mobility and gender is shown in Table 2 (univariate analyses) and in Table 3 (multivariate analyses). Figures 1 (BMI) and 2 (WC) show the trajectory of BMI and WC by gender, social mobility and birth weight.

**Table 2:** Univariate analysis between BMI and WC according to birth weight and social mobility, 1978/79 Ribeirão Preto Birth Cohort

Social Mobility	Gender	Birth weight		P*	P**	P***	P****
BMI (kg/m^2^)		Mean (IC 95%)†					
		≥2500 g (*n* = 1880)	<2500 g (*n* = 123)				
High-High (*n* = 533)	Males Females	25.1 [24.6-25.7]^A^ 22.3 [21.8-22.9]^a,B^	25.2 [21.7-28.7] 24.6 [21.9-27.2]^ab^	0.074	0.190	**0.022**	**0.030**
Low-High (*n* = 1170)	Males Females	24.8 [24.4-25.2]^A^ 23.9 [23.5-24.3]^b,B,α^	24.7 [22.9-26.5]^A^ 22.1 [20.7-23.5]^a,B,γ^				
Low-Low (*n* = 300)	Males Females	25.4 [24.6-26.2] 24.7 [23.9-25.5]^b,α^	24.7 [22.5-27.0] 27.6 [25.4-29.9]^b,γ^				
WC (cm)		Mean (IC 95%)†					
		≥2500 g (*n* = 1885)	<2500 g (*n* = 123)				
High-High (*n* = 533)	Males Females	88.1 [86.7-89.5]^A^ 73.8 [72.3-75.2]^a,B^	86.6 [78.1-95.2] 77.8 [71.2-84.3]^ab^	0.100	0.082	**0.007**	**0.017**
Low-High (*n* = 1172)	Males Females	87.2 [86.2-88.2]^A^ 77.4 [76.5-78.4]^b,B,α^	86.2 [81.8-90.5]^A^ 73.1 [69.6-76.6]^a,B,γ^				
Low-Low (*n* = 303)	Males Females	88.8 [86.8-90.7]^A^ 79.6 [77.6-81.5]^b,B,α^	85.7 [80.2-91.2] 87.5 [82.0-93.0]^b,γ^				

*P* refers to three-way ANOVA (Bonferroni *post hoc* pairwise comparison)

†Value expressed as mean and 95% confidence interval

Different small letters denote statistically significant differences according to gender and birth weight and by social mobility (*P* < 0.05)

Different capital letters denote statistically significant differences according to social mobility and birth weight and by gender (*P* <0.05)

Different Greek letters denote statistically significant differences according to social mobility and gender and by birth weight (*P* < 0.05)

*P: outcomes *vs.* interaction between social mobility and birth weight

**P: outcomes *vs.* interaction between gender and birth weight

***P: outcomes *vs.* interaction between social mobility and gender

****P: outcomes *vs.* interaction between social mobility, birth weight and gender

*BMI* body mass index, *WC* waist circumference

Bold text: Significant differences between groups (*P* < 0.05)

**Table 3:** Adjusted analysis between BMI and WC according to birth weight and social mobility, 1978/79 Ribeirão Preto Birth Cohort

Social Mobility	Gender	Birth weight		P*	P**	P***	P****
BMI (kg/m^2^)		Mean (IC 95%)†					
		≥2500 g (*n* = 1532)	<2500 g (*n* = 86)				
High-High (*n* = 471)	Males Females	25.4 [24.8-26.0]^A^ 22.5 [21.9-23.1]^a,B^	26.1 [22.4-29.8] 24.3 [21.4-27.1]^ab^	0.125	0.277	**0.017**	**0.044**
Low-High (*n* = 931)	Males Females	25.1 [24.7-25.6]^A^ 23.9 [23.5-24.4]^b,B,α^	24.6 [22.2-27.0]^A^ 21.6 [20.0-23.2]^a,B,γ^				
Low-Low (*n* = 216)	Males Females	26.1 [25.2-27.0] 25.1 [24.1-26.0]^b^	24.1 [21.7-26.5] 27.5 [24.8-30.2]^b^				
WC (cm)		Mean (IC 95%)†					
		≥2500 g (*n* = 1536)	<2500 g (*n* = 86)				
High-High (*n* = 471)	Males Females	88.4 [86.8-89.9]^A^ 74.1 [72.6-75.7]^a,B^	89.6 [80.4-98.7]^A^ 76.8 [69.7-83.9]^ab,B^	0.311	0.285	**0.009**	**0.035**
Low-High (*n* = 933)	Males Females	87.9 [86.8-89.1]^A^ 77.3 [76.2-78.5]^b,B,α^	86.9 [80.9-92.9]^A^ 72.2 [68.2-76.2]^a,B,γ^				
Low-Low (*n* = 218)	Males Females	90.2 [88.0-92.5]^A^ 80.4 [78.0-82.7]^b,B^	84.1 [78.1-90.1] 86.0 [79.2-92.7]^b^				

*P* refers to Analysis of Covariance Models (ANCOVA) (Bonferroni *post hoc* pairwise comparison)

Model adjusted for: smoking during pregnancy, type of delivery, gestational age (39.3 weeks)

†Value expressed as mean and 95% confidence interval

Different small letters denote statistically significant differences according to gender and birth weight and by social mobility (*P* < 0.05)

Different capital letters denote statistically significant differences according to social mobility and birth weight and by gender (*P* < 0.05)

Different Greek letters denote statistically significant differences according to social mobility and gender and by birth weight (*P* < 0.05)

*P: outcomes *vs.* interaction between social mobility and birth weight

**P: outcomes *vs.* interaction between gender and birth weight

***P: outcomes *vs.* interaction between social mobility and gender

****P: outcomes *vs.* interaction between social mobility, birth weight and gender

*BMI* body mass index, *WC* waist circumference

Bold text: Significant differences between groups (*P* < 0.05)

**Fig. 2:**
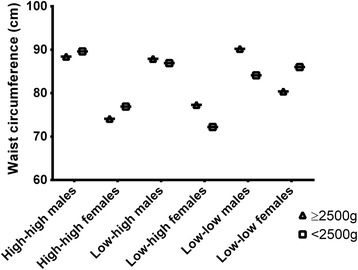
Distribution of mean WC values (females *vs.* males) according to social mobility and birth weight

There were no statistically significant interactions between the outcomes, social mobility and birth weight (*P* > 0.05) or between gender and birth weight (*P* > 0.05). However, in the crude and adjusted model, there was a statistically significant interaction between social mobility and gender (*P* < 0.05) and a triple interaction between social mobility, birth weight and gender (*P* < 0.05).

The biological values of males were significantly higher than those of females, specifically regarding BMI, as follows: High-High group with no LBW (values: 22.3 kg/m^2^ *vs*. 25.1 kg/m^2^), Low-High group with no LBW (values: 23.9 kg/m^2^ *vs*. 24.8 kg/m^2^) and Low-High group with LBW (values: 22.1 kg/m^2^ *vs*. 24.7 kg/m^2^). The same statistical differences were maintained in adjusted analyses. Also about WC, the male values were significantly higher than the female values, as follows: High-High group with no LBW (values: 88.1 cm *vs*. 73.8 cm), Low-High group with no LBW (values: 87.2 cm *vs*. 77.4 cm), Low-Low group with no LBW (88.8 cm *vs*. 79.6 cm) and Low-High group with LBW (values: 86.2 cm *vs*. 73.1 cm). The same statistical differences were maintained in adjusted analyses and the values of WC in High-High group with LBW (values: 89.6 cm *vs*. 76.8 cm) were higher in males than females. Gender differences are indicated by capital letters in Tables 2 and 3.

Regarding differences in social mobility, crude analysis showed that among females born without LBW, BMI and WC were statistical higher in the Low-Low group (values: 24.7 kg/m^2^ and 79.6 cm) compared to the High-High group (values: 22.3 kg/m^2^ and 73.8 cm). Among LBW women, BMI and WC were significantly higher in the Low-Low group (values: 27.6 kg/m^2^ and 87.5 cm) compared to the Low-High group (values: 22.1 kg/m^2^and 73.1 cm). The same statistical differences were maintained in adjusted analyses. No statistically significant differences were found for males (*P* > 0.05). The differences in social mobility are indicated by small letters in Tables 2 and 3.

Regarding the differences in birth weight (no LBW *vs.* LBW), only among females, BMI and WC of the Low-High group were higher in non LBW (values: 23.9 kg/m^2^ and 77.4 cm) than LBW (values: 22.1 kg/m^2^ and 73.1 cm) subjects. However, BMI and WC of the Low-Low group were higher in LBW (values: 27.6 kg/m^2^ and 87.5 cm) when compared to non LBW (values: 24.7 kg/m^2^ and 79.6 cm) subjects. The significant difference of the Low-High group was maintained in adjusted analyses. The differences in birth weight are indicated by Greek letters in Tables 2 and 3.

## Discussion

The present results show that the metabolic consequences of social mobility are especially pronounced among adult women. There was a triple interaction between social mobility, birth weight and gender. Among LBW women, BMI and WC were significantly higher for those who did not present social mobility and who stayed in the low schooling group from birth to early adulthood. Conversely, LBW women under social mobility showed the lowest BMI and WC, changing prognoses with a rapid response to the social and possible environmental changes. Moreover, the BMI and WC values of those with upward socioeconomic mobility (Low-High) were higher among women without LBW than among women with LBW. The results pointed out that the scenario characterized by social scarcity and lack of conditions can be overcome by social improvement [31].

Other longitudinal studies have assessed the relationship between SES mobility and metabolic outcomes. For example, a study conducted in Cuiaba, Brazil, with 1716 individuals, found that the higher SES group (based on the number of home appliances, cars and paid maids, and the educational level of the head of the household and compared between pre-school and adolescence) at pre-school age and among those who remained in the high SES until adolescence was positively associated with overweight between birth and adolescence [32]. In contrast, a study of United States (US) immigrants that investigated the associations between SES mobility categories (based on the educational attainment reported by individuals as adults and by their parents during adolescence) found that the upward educational mobility, compared to maintaining a low SES, was associated with a lower mean BMI [33]. Thus, these results showed a social determinant of changes in metabolic aspects and could indicate the stage of economic development of different cities. In this investigation, in contrast to the Cuiaba study and in agreement with the US study, upward social mobility protected against obesity. However, stability in the low schooling group was associated with higher BMI and WC values only among women born with LBW. However, being born in a low schooling environments and remaining with low schooling during early adult life had no effect on obesity among men or among those born LBW.

The biological explanation for the association between socioeconomic mobility and health outcomes may involve changes in stress-related processes and the cardiovascular system which is one of the most susceptible systems to stress [34]. A recent cohort study conducted in South Africa showed that an upward SES change (measured with a physical asset-based tool for the determination of household socioeconomic status) between infancy and adolescence was significantly associated with lower systolic blood pressure at the age of 18 years, compared to a persistent low SES [35].

Furthermore, we also found that higher BMI and WC values are especially pronounced among adult women born LBW (Low-Low group). However, in the Low-High group, BMI and WC values were higher in non LBW subjects compared to LBW subjects. Another population-based cohort investigating the effect of family background and birth region on adult obesity status by gender and over the life course found that birth region and mother’s education were associated with an increased obesity risk among women, but not among men [36].

In addition, the WC of the High-High group with LBW was higher among males than among females. Other studies conducted in Brazil also seem to agree about a higher risk of obesity among men who have always been in the higher socioeconomic strata, whereas the opposite has been found for women [16, 37, 38]. These findings suggest that women are experiencing the social transition (or reversal of the social gradient) much faster than men [16].

The plausible mechanisms underlying the association between an early life stress (LBW) and obesity in adulthood may involve a rapid rate of weight gain in early life or a combination of LBW and rapid postnatal catch-up growth [39]. A prospective cohort study conducted in Australia showed that rapid weight gain in the first years of life increased the risk of a higher BMI and WC in young adulthood. The authors found that the rapid growers had a significantly higher gestation period and LBW rate when compared to those who exhibited gradual or slow growth. In addition, the rate of weight gain was significantly associated with maternal education. Regarding gender differences, boys showed a higher growth rate than girls; however, when obesity status was classified by WC measurements at 21 years, more females were overweight than males [40].

Another explanation could be the long-term health implications of caesarean section (CS) delivery [41, 42] and the effect of changes in the gut microbiota [43–45]. Data from a Brazilian study obtained from SINASC (Brazilian Live Births Database) suggest a non-linear trend association. When CS rates were about 30%, LBW rates tended to decline as CS increased. On the other hand, when the rates were higher than 30%, LBW rates tended to increase with CS [18]. However, CS rates are lower in private social groups, and therefore the results do not support this hypothesis. In addition, the interaction between type of delivery and maternal BMI is also important, with maternal obesity and CS having been associated with changes in the microbiota of newborns. Unfortunately, we do not have information about maternal BMI in order to explore its impact on offspring BMI and WC. Moreover, there are many environmental determinants and psychological factors involved to the predictors of obesity which mediate the association with LBW, such as maternal cigarette smoking, maternal education and inadequate maternal nutrition [46]. Regarding gender differences in social patterning, social childhood exposure seems to influence later life outcomes, including biological programming during critical periods of growth and the early acquisition an unhealthy lifestyle [47]. Furthermore, this gender effect on outcome can be modified by birth weight.

Regarding the different metabolic outcomes between genders, a study of the same cohort in Ribeirão Preto, São Paulo, Brazil, revealed that the association between BMI at birth and adulthood BMI occurred only among women, suggesting that prevention policies should consider gender-specific strategies [48]. Moreover, in another birth cohort, the authors found differences between men and women: BMI was lower in men of low SES than in men of high SES but was higher in women of low SES than in women of high SES [49]. These different gender results may be explained by differences in *in utero* programming epigenetic mechanisms in addition to health and feeding behavior. In a review explaining the sex-specific adaptation of causal variables to environmental perturbations, when the sex of the embryo was taken into account. Most human studies showed sexual dimorphism in placental function and a specific sexually dimorphic response to maternal diet [50]. Using data from a township in South Africa, the authors observed that women who were nutritionally deprived as children are more likely to be obese as adults, whereas men who were deprived as children appear to face no greater obesity risk [51]. The contribution to obesity can be justified by the fact that women from a Brazilian birth cohort, especially those born with severe intrauterine growth restriction, measured by the birth weight ratio, preferred carbohydrates to protein in their regular diet [52].

The effects of social determinants on total and central obesity agree with theories of disease etiology involving fetal programming and their interactions according to gender and should be investigated more fully [8]. To investigate the effect of neighborhood changes on metabolic outcomes, a randomized social experiment involving a total of 4498 families showed that the prevalence of extreme obesity and diabetes decreased with the opportunity to move from a neighborhood with a high level of poverty to a less underprivileged one [31]. Complementarily, a study with the same design also found that neighborhood environments affects the well-being of low-income adult [53].

Regarding study limitation, in the cited birth cohort, no information was obtained about maternal obesity (measured by BMI or body composition). In contrast, the size of our birth cohort is relatively large and the data were collected during transition periods.

## Conclusions

In summary, the present results indicate that, among females, the stable Low-Low schooling group had higher BMI and WC values, suggesting that the individuals maintained a low schooling level, with less access to health services, to the conditions needed to maintain a healthy lifestyle and to knowledge of issues related to disease prevention. However, being born with LBW in a “low schooling” environment and achieving high educational level (Low-High group) was associated with less detrimental metabolic features. On the other hand, not being born LBW in a “high schooling” environment, achieving a high educational level (High-High group) was associated with less detrimental metabolic features. Essentially, we found that social mobility does not affect total (BMI) and central obesity (WC) in the same direction.

## Abbreviations

ANCOVA: Analysis of covariance
ANOVA: Analysis of variance
BMI: Body mass index
CNPq: Brazilian Research Council
CS: Caesarean section
LBW: Low birth weight
SD: Standard deviation
SES: Socioeconomic status
SINASC: Brazilian Live Births Database
SPSS: Statistical Package for the Social Science
US: United States
WC: Waist circumference
